# A novel biomarker for prediction of atrial fibrillation susceptibility in patients with celiac disease

**DOI:** 10.1371/journal.pone.0190382

**Published:** 2018-01-09

**Authors:** Selcuk Kucukseymen, Ayhan Hilmi Cekin, Nermin Bayar, Sakir Arslan, Elif Uygur Kucukseymen, Tanju Mercan, Semir Ozdemir

**Affiliations:** 1 Department of Cardiology, University of Health Sciences, Antalya Education and Research Hospital, Antalya, Turkey; 2 Department of Biophysics, Akdeniz University Faculty of Medicine, Antalya, Turkey; 3 Department of Gastroenterology, University of Health Sciences, Antalya Education and Research Hospital, Antalya, Turkey; 4 Department of Neurology, University of Health Sciences, Antalya Education and Research Hospital, Antalya, Turkey; Indiana University, UNITED STATES

## Abstract

**Background:**

Celiac disease (CD), a serious autoimmune disorder that occurs in people who are genetically predisposed, is induced by dietary gluten intake and affects primarily the small intestine. Many studies have identified an increased risk of cardiovascular problems in patients with CD. Moreover, these patients are susceptible to certain liver diseases, as well as fibrosis.

**Objective:**

The aim of this study was to assess the presence of fibrosis using the De Ritis ratio, determining its effect on the electromechanical features of the left atrium and its susceptibility to atrial fibrillation (AF) in patients with CD.

**Methods:**

A total of 97 patients diagnosed with CD by antibody test and biopsy were included in this prospective study. Two groups were created from these patients, a fibrosis-prone (FP) group and a non-fibrosis-prone (NFP) group, according to the cut-off value, as defined in previously published reports, for the AST/ALT ratio. Electrocardiographic and echocardiographic examinations were performed as part of the study.

**Results:**

There were no differences in the baseline characteristics and conventional echocardiographic parameters of the defined groups. However, the patients in the FP group, as compared to those in the NFP group, had significantly increased PWD (56.68±6.48 ms vs. 37.49±6.22 ms, P<0.001). Additionally, significantly higher interatrial (60.50±13.05 ms vs. 29.40±11.55 ms, P<0.001), intra-left atrial (44.18±14.12 ms vs. 21.02±11.99 ms, P<0.001), and intra-right atrial (15.61±8.91 ms vs. 8.38±4.50 ms, P<0.001) EMD was found among the patients in the FP group compared to that of the NFP group.

**Conclusion:**

It is believed that the susceptibility to AF cited in previous studies may be related to fibrosis. Our study is the first to examine the possible effects of fibrosis on AF susceptibility in patients with CD, whereby we propose a new biomarker for prediction of AF susceptibility of these patients.

## Introduction

Celiac disease (CD) is a multisystem, chronic immune-mediated disorder that affects several organs [1]. CD causes a number of gastrointestinal problems, such as diarrhea, abdominal distention and malabsorption, due to the intake of grain proteins, which are collectively referred to as a “gluten” diet [1, 2]. Cardiovascular symptoms, like arrhythmias or irregular heartbeats, coronary artery diseases, and possible heart failure, have also been also reported to be associated with CD [2–6]. Some studies have indicated that these symptoms are caused by inflammation, while others have suggested the involvement of the autoimmune system response [7–10]. CD patients also have a higher susceptibility to liver diseases, such as non-alcoholic fatty liver disease (NAFLD) [11]. Here it is important to note that NAFLD and other liver diseases may progress to cirrhosis of the liver, and fibrosis is the basic pathophysiological mechanism for cirrhosis [12, 13].

In the literature, there are five scoring systems that are used to demonstrate fibrosis in liver diseases. These include the aspartate aminotransferase/alanine aminotransferase (AST/ALT) ratio, the AST to platelet ratio index, and the BARD, FIB-4 and NAFLD fibrosis scores [14–18]. Among these, the AST/ALT ratio (AAR), also referred to as the De Ritis ratio, is particularly valuable, as it is a non-invasive, easy and reliable scoring system for the determination of fibrosis [16, 19]. In studies conducted on patients with high AAR scores, fibrosis, and consequently cirrhosis rates, were shown to be significantly higher [16, 19].

The development of atrial fibrillation (AF) in patients with CD has been shown to occur at a higher rate than that of normal, healthy individuals [3, 20], with inflammation having been suggested as the potential mechanism of increased AF susceptibility in patients with CD [7, 8, 10]. In this study, given that the fibrosis pathway has been identified as an important pathophysiological mechanism for the development of AF [21, 22] in the general population, we examine the interaction between fibrosis and AF susceptibility in CD patients. Thus, we sought to determine whether AAR, which is a fibrosis marker, can be used, as an alternative to inflammation markers, for the prediction of increased AF risk in patients with CD. Our study is the first to examine the fibrosis effects on AF in patients with CD, and thereby, to show the correlation of AAR with AF risk.

## Materials and methods

### Patient selection

A total of 105 consecutive patients with serology (plasma anti-tissue transglutaminase and anti-endomysial antibodies) and biopsy-proven CD were recruited from the University of Health Sciences, Antalya Education and Research Hospital, Gastroenterology clinic. Patients were excluded if they had any other liver diseases or gastrointestinal diseases, diabetes mellitus, kidney diseases, or thyroid dysfunction, or if they consumed alcohol. From the study population, 3 patients were excluded due to DM (HbA1c >6%), 2 patients due to having an active infection (CRP >3.15 mg/dl), 1 patient due to thyroid dysfunction (outside of normal limits of T4 and T3 hormones), and 2 patients due to alcohol consumption (as discovered from a questionnaire). Therefore, the study began with 97 patients. Ethical approval was obtained from the University of Health Sciences, Antalya Education and Research Hospital Ethics Committee. In addition, the patients signed consent forms after being provided information about the study, including its purpose.

The CD-diagnosed patients were divided into two groups according to the cut-off point of 0.8 for AAR, which has been established in previously published reports [17]. Patients whose cut-off value was higher than 0.8 were placed in the fibrosis-proven group (FP), while the others were assigned to the non-fibrosis-proven (NFP) group. The FP group consisted of 44 (56.1% female) patients, and the NFP group, 53 (52.83% female) patients. Heart rate and blood pressure were measured in all patients.

### Electrocardiographic examination

A 12-lead electrocardiography (ECG) recording (Nihon Kohden, Tokyo, Japan) was performed on the patients in both groups. The onset of the P wave was defined as the point of first visible upward slope from baseline for positive waveforms and as the point of first downward slope from baseline for negative waveforms. The return to the baseline was considered the end of the P wave. The maximum P wave duration (P_max_), measured by hand on paper in all of the 12 leads, was used as the longest atrial conduction time. In contrast, the minimum P wave duration (P_min_), measured by hand on paper in all of the 12 leads, was used as the shortest atrial conduction time. PWD was defined as the difference between the P_max_ and the P_min_ [23].

### Echocardiographic examination

**Fig 1:**
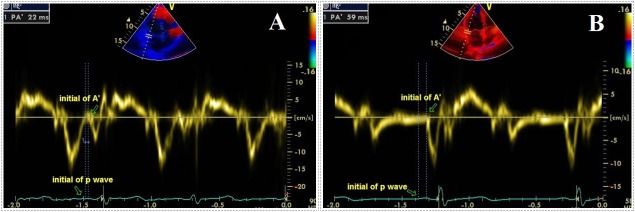
**Atrial electromechanical coupling (PA’)**; the time interval from the onset of the P-wave on the surface electrocardiogram to the beginning of the late diastolic wave A’ [in a patient with CD (A) and normal individual (B).

All patients underwent 2-dimensional, M-mode, pulsed, and color flow Doppler echocardiographic examinations (Philips EPIQ 7 Cardiac Ultrasound, Bothell, WA, USA). During echocardiography, a single-lead electrocardiogram was recorded simultaneously. Data were recorded from the average of three cardiac cycles. Next, a tissue Doppler echocardiography, with transducer frequencies of 3.5–4.0 MHz, was performed by adjusting the spectral pulsed- Doppler signal filters until a Nyquist limit of 15 to 20 cm/s was reached using the minimal optimal gain. The monitor sweep speed was set at 100 mm/s. In the apical four-chamber view, the pulsed-Doppler sample volume was placed at the level of LV lateral mitral annulus, septal mitral annulus, and RV tricuspid annulus. Atrial electromechanical coupling (PA), the time interval from the onset of the P-wave on the surface electrocardiogram to the beginning of the late diastolic wave (A), was obtained from the lateral mitral annulus (PA lateral), septal mitral annulus (PA septal), and tricuspid annulus (PA tricuspid) (Fig 1). The difference between PA lateral and PA tricuspid was defined as the interatrial electromechanical delay (EMD); the difference between PA septal and PA tricuspid was defined as the intra-right atrial EMD; and the difference between PA septal and PA lateral was defined as the intra-left atrial EMD [24, 25].

### Measuring laboratory parameters

Routine biochemical tests and complete blood count (CBC) were performed from antecubital venous sampling after 12 hours of fasting. The blood samples were centrifuged and serum samples were collected. The CRP levels (normal range: 0–5 mg/L) were analyzed with a Beckman Coulter analyzer. (Image 800; Fullerton, CA, USA). CBC, including white blood cell (WBC), neutrophil, and lymphocyte counts, was performed using an automated CBC device (Abbott Cell-Dyn, Abbott Park, IL, USA). The neutrophil/ lymphocyte ratio (NLR) was calculated using data obtained from the CBC.

### Statistical analysis

Statistical evaluations were performed using SPSS 24 for Windows (SPSS INC., Chicago, IL, USA). All results were first subjected to a normality test, and once passed; they were analyzed by the Shapiro-Wilk test and Student t test. A P value of < 0.05 was considered significant. Mann-Whitney U-test was used for non-normally distributed variables. Variables were described as mean ± standard deviation, and nominal variables were expressed as percentage. The chi-square test was used for nominal variables. To compare the groups, Student’s t-test for mean value and box-and-whisker plot for median, maximum and minimum values of left EMD, right EMD and inter-atrial EMD were performed. Next correlation between EMD values and AST/ALT ratios was analyzed and R values were determined for each region of atria by Spearman’s correlation test. P values less than 0.05 were considered statically significant.

## Results

A total of 97 patients with CD participated in this prospective study. The baseline characteristics of both groups are shown in Table 1. In comparing both groups, there were no statistically significant differences found regarding age, gender, percentage of patients who smoked, body mass index, hyperlipidemia, high sensitive C reactive protein (hsCRP), NLR, total bilirubin, gamma glutamyl transferase (GGT), alkaline phosphatase (ALP) and blood pressure (p>0.05).

**Table 1:** Demographic characteristics of the study populations.

	NFP group(n = 53)	FP group(n = 44)	P value
**Age.years**	43.96±11.52	40.43±10.86	0.124
**Female.n(%)**	52.83	56.10	0.957
**Smoking.n(%)**	33.96	40.90	0.531
**BMI (kg/m**^**2**^**)**	23.7±4.35	24.09±2.07	0.608
**Total cholesterol (mg/dL)**	200±47.6	188.14±42.11	0.192
**LDL (mg/dL)**	129±41.2	116.81±36.86	0.135
**HDL (mg/dL)**	43.7±9.24	42.91±11.55	0.728
**Triglyceride (mg/dL)**	138±62.8	141.57±53.98	0.796
**Uric acid (mg/dL)**	4.58±1.54	4.18±1.22	0.157
**CRP (mg/L)**	2.18±0.95	2.05±1.16	0.549
**N/L ratio**	2.65±0.69	2.68±0.67	0.827
**Hemeoglobin (g/dL)**	12.86±1.78	13.10±1.59	0.501
**WBC (10^3/mm**^**3**^**)**	7.81±1.35	7.86±1.84	0.876
**Total bilirubin (mg/dL)**	0.71±0.28	0.73±0.24	0.711
**GGT (U/L)**	24.75±8.94	23.73±7.89	0.549
**ALP (U/L)**	70.68±25.42	70.45±27.05	0.967
**SBP (mm Hg)**	127.25±8.05	124.75±10.57	0.202

BMI = body mass index; HDL = high-density lipoprotein; LDL = low-density lipoprotein; CRP = C-reactive protein; N/L = neutrophil/lymphocyte; WBC = White blood cell; GGT = gamma glutamyl transferase; ALP = alkaline phosphatase; SBP = Systolic blood pressure; DBP = diastolic blood pressure. The values show a normal distribution mean ± SD

Among the conventional and tissue Doppler echocardiographic parameters, the left ventricular (LV) ejection fraction, interventricular septum thickness, posterior wall thickness, left atrial diameter, LV end-diastolic diameter, LV end-systolic diameter and LV diastolic function parameters, such as LV E/A ratio and LV isovolumetric relaxation time, were determined to be similar in the patients from both groups in Table 2.

**Table 2:** Conventional echocardiographic parameters of the study populations.

Variables	NFP group(n = 53)	FP group(n = 44)	P value
**LVEF (%)**	63.81±2.42	64.80±1.84	0.125
**LA diameter (mm)**	30.51±3.94	31.89±4.09	0.097
**LVEDD (mm)**	41.55±4.38	43.11±4.70	0.095
**LVESD (mm)**	23.98±2.71	24.66±4.10	0.371
**E/A ratio**	1.22±0.46	1.34±0.45	0.201
**E/E’**	0.64±0.15	0.67±0.29	0.485
**EDT (ms)**	208.72±29.92	211.68±41.78	0.695
**IVRT (ms)**	85.51±15.29	84.41±12.40	0.696
**LVH presence.n(%)**	3.77	4.54	0.852

EDT = E-wave deceleration time; IVRT = isovolumic relaxation time; LA = left atrium–parasternal long axis; LVEF = left ventricular ejection fraction; LVEDD = left ventricular end-diastolic diameter; LVESD = left ventricular end-systolic diameter; LVH = left ventricular hypertrophy. The values show a normal distribution mean ± SD

**Fig 2:**
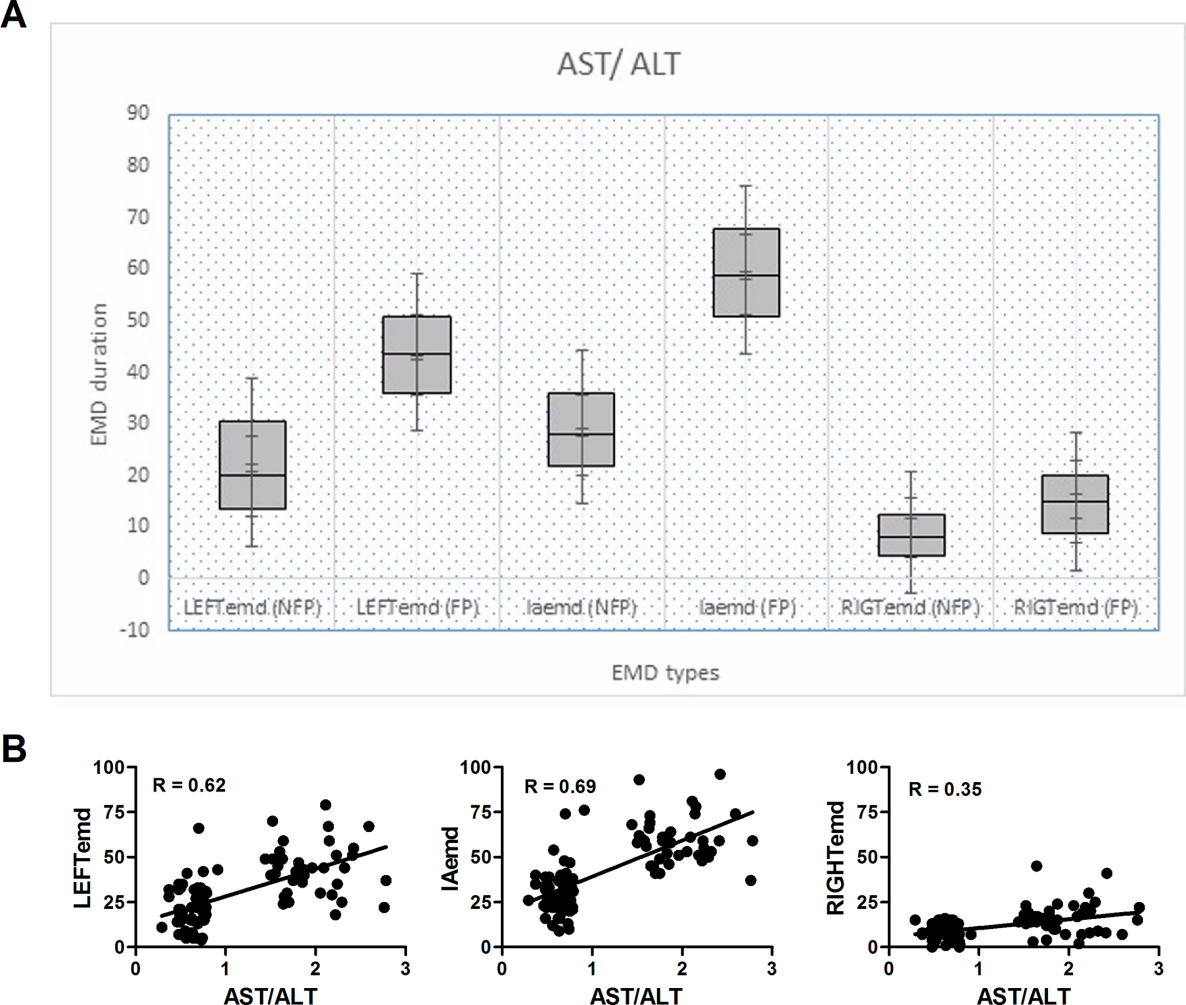
Relationship between atrial EMD values and fibrosis. (**A**) Differences between each EMDs in NFP and FP groups have been clearly shown in the box and whisker diagram. (**B**) Correlation analysis between atrial EMD values and AST/ALT ratio (AAR).

Analyses performed to assess the electrical functions of the left atrium showed there to be a higher PWD (56.68±6.48 ms vs. 37.49±6.22 ms, P < 0.001) in the FP group than that in the NFP group. On the other hand, PA lateral, PA septal, and PA tricuspid (P < 0.001 for all) were significantly increased in FP group patients as compared to NFP group patients. Furthermore, FP group patients had significantly higher intra-left atrial EMD (44.18±14.12 ms vs. 21.02±11.99 ms, P<0.001), intra-right atrial EMD (15.61±8.91 ms vs. 8.38±4.50 ms, P<0.001) and interatrial EMD (60.50±13.05 ms vs. 29.40±11.55 ms, P<0.001) in Table 3. These differences are clearly shown for each EMD in a box and whisker plot graph. However the correlation between EMD values and AST/ALT ratio of right atria was relatively low while it was significantly higher for left atria and inter-atrial measurements (Fig 2).

**Table 3:** Atrial electrical activity parameters of the study populations.

Variables	NFP group(n = 53)	FP group(n = 44)	P values
**P**_**min**_ **(ms)**	52.73±6.63	57.75±6.60	*P*<0.001*
**P**_**max**_ **(ms)**	95.25±8.92	109.41±8.92	*P*<0.001*
**PWD (ms)**	37.49±6.22	56.68±6.48	*P*<0.001*
**PA’**_**septal**_ **(ms)**	81.94±11.67	75.82±12.85	*P*<0.001*
**PA’**_**laterall**_ **(ms)**	102.96±13.27	119.32±15.04	*P*<0.001*
**PA’**_**tricuspid**_ **(ms)**	73.57±10.75	60.09±13.64	*P*<0.001*
**Intra-LA-EMD (ms)**	21.02±11.99	44.18±14.12	*P*<0.001*
**Intra-RA-EMD (ms)**	8.38±4.50	15.61±8.91	*P*<0.001*
**Interatrial-EMD (ms)**	29.40±11.55	60.50±13.05	*P*<0.001*

PWD = P-wave dispersion; LA = left atrium; RA = right atrium; EMD = electromechanical delay

“*” = A statistically significant difference. The values show a normal distribution mean ± SD

## Discussion

CD is a chronic inflammatory disease of the gastrointestinal system that is characterized by chronic malabsorption in sensitive individuals who ingest grains containing gluten [1, 26]. The worldwide prevalence of CD is 1% in the general population, but in patients with autoimmune disorders the prevalence is higher than that of the normal population (8–20%) [1, 2]. Small intestinal villous atrophy and crypt hyperplasia are the main pathological findings in pediatric cases, whereas, lymphocytic infiltration, with or without the aforementioned symptoms, is more common in the adult form of the disease [1, 27].

Recent studies have shown that patients with CD may have a higher prevalence of risk factors for cardiovascular diseases (CVD), such as arrhythmia, atherosclerotic coronary disease and heart failure, compared to the general population. While the CVD risk factors are not well defined in CD[3, 4, 26, 28, 29], the risk of atrial fibrillation is the most pronounced among these factors [3, 20]. Consistent with this, in a study conducted by Emilsson et al., a significantly higher atrial fibrillation (AF) risk than that seen in the normal population was reported in CD patients [3]. Furthermore, in another study by Emilsson et al., a remarkable increase in tendency toward dilated cardiomyopathy and cardiovascular death, as well as in AF risks, in patients with CD was reported [30]. Likewise, we too found increased AF risk in CD patients [20]. This increased risk of AF has been attributed primarily to the increase of inflammatory markers and autoimmunity in previously published studies.

It is well-known that CD patients have increased risk for AF, but in this study, the fundamental aim was to identify which CD patients in particular are at greater risk. Therefore, this study did not include any AF-diagnosed patients. However, in CD patients, there is the same degree of risk of AF, or paroxysmal AF, which is one of the AF forms [31]. There are different methods for predicting AF risk [31, 32]. Although most of the related studies conducted have aimed to detect the predictors of AF recurrence, none of the parameters have been able to predict with 100% accuracy [33]. New parameters, therefore, continue to be investigated for predicting the recurrence of AF precisely. Electrocardiography (ECG) and transthoracic echocardiography (TTE) are commonly used to carry out these investigations [24, 25, 34, 35]. In ECG, P wave dispersion (PWD) constitutes a recent contribution to non-invasive electrocardiology [23, 34]. As this electrocardiographic measurement reflects a disparity in atrial conduction, it has been proposed as a predictor for AF in studies on PWD [23]. Similarly, in our study, we calculated all PWD values for prediction of AF risk in the ECG of patients. Patients in the FP group had a higher rate of PWD, which implied higher arrhythmia susceptibility.

Another commonly used estimation parameter is the tissue Doppler echocardiography (TDI), which is one of the TTE procedures. With the TDI, the left atrial electromechanical delay (EMD) times can be calculated [20, 24, 25]. Relevant to this, in studies related with EMD, such as those conducted by Ari et al. and Akil et al., EMD is commonly suggested as a useful parameter for prediction of AF onsets and relapses [25]. There are three EMD types; interatrial, intra-left and intra-right [24]. The following EMD parameters can be calculated: atrial electromechanical coupling (PA’), and the time interval from the onset of the P-wave on the surface electrocardiogram to the beginning of the late diastolic wave A’ [23]. As the prediction of AF is also important for determining risk of stroke, these methods are actively used for the evaluation of stroke patients, and if this risk is found to be high, cardioembolic events should be examined. In our study, we calculated all EMD parameters for prediction of AF risk in both groups. As with the ECG findings, we found all the EMD parameters to be higher in the FP group, which may infer high susceptibility to fibrosis risk.

Current findings suggest that fibrosis and inflammation are important risk factors for AF [7–9, 21, 22]. However, in CD patients, AF risk is associated primarily with inflammation that presents with significant increase in CRP, IL6, and TNF-alpha [6, 27, 36]. Numerous studies, including those by Aviles et al., Hu et al., and Boos et al., have examined the correlation between inflammation and AF risks in detail, and as result, the association between inflammation and AF has been unequivocally confirmed [7, 10]. On the other hand, the fibrosis pathway can also serve as an important factor for predicting this risk [17, 22]. Considering this, we performed an extensive review of the literature to learn more about the relationship between fibrosis and CD. Interestingly, we found strong evidence to infer a connection between non-alcoholic fatty liver disease (NAFLD) and CD, a connection which has been discussed comprehensively by Reilly et al. [11]. According to previous studies, CD is also associated with different liver diseases, some of which are related to fibrosis, including hepatobiliary disorders, asymptomatic elevations of liver enzyme levels, NAFLD, autoimmune hepatobiliary disorders, autoimmune hepatitis and cholangitis, primary sclerosing cholangitis, primary biliary cirrhosis, miscellaneous hepatobiliary disorders in CD, hepatic vein obstruction, noncirrhotic portal hypertension, and end-stage liver disease. Considering that NAFLD is known to be a secondary disease in the pathology of cirrhosis [12, 13], we decided to examine fibrosis parameters in CD patients. There are numerous non-invasive methods that can be used for the prediction of fibrosis, including the AST/ALT ratio (AAR), APRI, FIB4 and the NAFLD fibrosis score [13, 14, 16–18]. For the aim of the study, it was important that we choose a parameter that has been used in predicting cardiovascular diseases [19, 37–41]. Therefore, we used AAR for the prediction of fibrosis risk in CD patients. Next, we used the cut-off value of 0.8 for AAR, which was based on recently published, large-scale trials, like the Framingham Heart Study [14]. The CD patients were then divided into two groups according to their cut-off value for AAR. Patients with a value ≥ 0.8 were placed in the FP-designated group, while the others were placed in the NFP-designated group. We went on to compare them according to selected parameters for prediction of AF risks. The results of this comparison allowed us to find the main reason for high AF risk and the method for predicting it in patients with CD.

While there are studies in the literature similar to our study [3, 20], our research is the first to make comparisons among CD patients. Although we know from the literature that CD patients are more susceptible to AF than the general population [20, 24], we propose an alternative tool for estimation of the probability of AF in CD patients. Moreover, our study included more patients than previous studies [20, 24] and serves as a subgroup study particularly pertaining to CD patients. According to our study results, CD patients are susceptible to fibrosis, and the fibrosis rate associated with this susceptibility can be obtained by determining AAR values. Otherwise stated, susceptibility to AF increases in patients who have high AAR values.

Some limitations of our study should be noted. This study is a single-center, nonrandomized study and the population was relatively small. The other limitation is that ultrasound/biopsy was not performed to examine the presence of liver diseases which is unknown for patients with normal enzyme level. Finally further studies included larger population scales are needed to show the accuracy of this hypothesis unequivocally.

## Conclusion

In the celiac disease population, there is the risk of cardiovascular diseases. EMD, which is a respected predictor of AF, increases in CD patients. However, the inflammation pathway may not be the only culprit for this delay. Fibrosis may be equally as responsible as the inflammation pathway. Our study is the first to demonstrate the fibrosis effects on AF susceptibility in patients with CD. These finding are clinically relevant particularly for CD patients with paroxysmal AF. However, large-scale prospective cohort studies conducted by multiple medical centers are strongly necessary to clarify the effectiveness of fibrosis and AAR value for prediction of AF susceptibility in patients with CD.

## Supporting information

S1 File All relevant data. (XLSX)
